# Online Interactive Flipped Classroom Teaching in Pediatrics for Medical Undergraduates

**DOI:** 10.7759/cureus.37603

**Published:** 2023-04-15

**Authors:** Jaya S Kaushik, Kausalya Raghuraman, Savita Verma, Vandana Arya, Virender K Gehlawat

**Affiliations:** 1 Pediatrics, All India Institute of Medical Sciences, Guwahati, IND; 2 Microbiology, All India Institute of Medical Sciences, Guwahati, IND; 3 Pharmacology, Pandit Bhagwat Dayal Sharma Post Graduate Institute of Medical Sciences, Rohtak, IND; 4 Pediatrics, Pandit Bhagwat Dayal Sharma Post Graduate Institute of Medical Sciences, Rohtak, IND

**Keywords:** increased engagement, student-centric teaching, seizure, pediatrics, online technology, medical education, flipped classroom

## Abstract

Objectives: To introduce online flipped classroom teaching for medical undergraduates in Pediatrics and to assess students' engagement and satisfaction with the students and faculty with the flipped classroom teaching method.

Methods: An interventional education study was conducted on online flipped classrooms for final-year medical undergraduates. The core team of faculty members was identified, students and faculty were sensitized, and pre-reading material and feedback forms were validated. Students were engaged using the Socrative app, and feedback from students and faculty was collected using Google Forms.

Results: One hundred sixty students and six faculty members participated in the study. During the scheduled class, 91.9% of students were engaged. The majority of the students strongly agreed that the flipped classroom was interesting (87.2%) and interactive (87%) and developed an interest in the subject of Pediatrics (86%). Faculty were also motivated to adopt this method.

Conclusion: The present study revealed that introducing flipped classroom strategy in an online model improved students' engagement and increased their interest in the subject.

## Introduction

There is a surge in online teaching following the coronavirus disease (COVID-19) pandemic. However, engaging the students during the entire online session was a significant challenge. The flipped classroom is a learner-centered educational model in which a course's lecture and homework elements are reversed [1]. A successful flipped classroom should allow the students to become critical thinkers, fully engage students and instructors, and stimulate the development of a deep understanding of the material [2]. The flipped classroom physical sessions have been evaluated among medical students, residents, doctors, nurses, or learners in other healthcare professions and disciplines (e.g., dental, pharmacy, environmental, or occupational health) [2-6]. 

Increased engagement in the class promotes active learning and improved capacity for analytical thinking by applying recently gained theoretical knowledge to clinical situations. However, with a surge in online classroom teaching, whether flipped classroom teaching can be executed online remains to be seen. Hence, the present study planned to introduce online flipped classroom interactive teaching modules in Pediatrics to undergraduate medical students and explore the perceptions of students and faculty.

## Materials and methods

It was a quasi-experimental study conducted among final-year medical undergraduate students from April to November 2020. The study was conducted in the Department of Pediatrics at a tertiary care hospital in India. The Biomedical Research Ethics Committee, Pandit Bhagwat Dayal Sharma Institute of Medical Sciences, approved the study with an approval number of BREC/20/79 (19.05.2020). The core team of faculty members was identified, consisting of three faculty members from Paediatrics and three medical education-trained faculty of the institute. A GANTT chart was prepared, teaching material, including pre-recorded lecture videos, and relevant articles and reading material were finalized. The core faculty members and external experts designed and validated feedback questionnaires for students and faculty members. The information regarding flipped classroom method of large group teaching was intimated to all students through institutional Google group email (Institutional G Suite). Students and faculty were sensitized to flipped classroom method online. A WhatsApp group (Whatsapp. Inc) was created for students and faculty to facilitate communication. Socrative App (downloaded from Google Play Store) was used to engage the students during the session.

The teaching material was provided to students one week before the online class. The pre-recorded lecture was uploaded to YouTube as an unlisted video, and the link was shared with students and faculty. Students were reminded to review the reading material and pre-recorded lectures before the class. The core team members and other departmental faculty of the institute who were sensitized joined the online session.

The scheduled online class time of one hour was utilized to discuss problem-based case scenarios. The online session was conducted using the institutional G suite per the expected lecture plan. A set of 12 questions were discussed during the session. These questions were clinical case scenario-based vignettes. A patient video accompanied a few questions to depict the clinical case scenario. Each case vignette was followed by a question students answered using the Socrative student app. The Socrative teacher app was used to record their responses. In each question, once the student clicked on the response, it would indicate whether the answer was right or wrong. Once students finished their responses, the investigator discussed the answer, and the learning message from that case scenario was explained to them before proceeding to the other case vignette. Similarly, students were engaged for the entire hour using 12 questions. No alteration or additional time was required for conducting these flipped classroom sessions.

Students' responses and involvement in the interactive platform determined their engagement. The data collected was anonymous, and an attempt to answer the question was considered an engagement. Students' perceptions and feedback were obtained. Knowledge assessment was assessed during the online session by recording the number of correct responses. A separate assessment test was not conducted to evaluate the gain in knowledge. Pre-validated feedback was obtained from the faculty and students using a Google form questionnaire.

The data were recorded in Microsoft Excel, including the student's engagement during the session (Socrative), student feedback, and faculty feedback. Student engagement during the online session and the number of correct responses were expressed as numbers and proportions. The student's and faculty's feedback were recorded as a Likert scale from one to five and described as numbers and ratios. The open-ended responses of the students were qualitatively analyzed using thematic analysis. The illustrative quotes were coded, subthemes identified, and themes were subsequently identified. 

## Results

**Figure 1:**
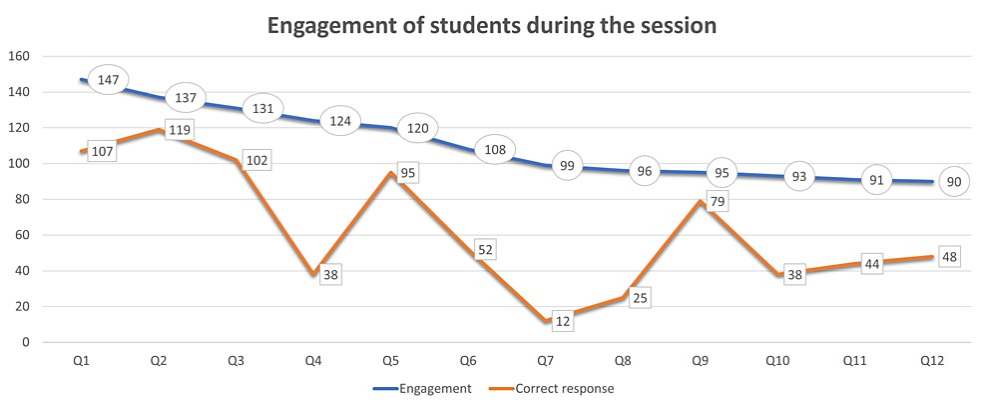
Engagement of students and many students with a correct response from questions (Q) 1 to 12 in the class as assessed by the Socrative app (n=160).

A total of 160 students and six faculty members participated in the study. There were 180 views for the YouTube pre-recorded video on the day of the scheduled online class. Although, after the class, the number of views increased to 240 for the same lecture. A total of 160 (91.4%) students attended the online class. The feedback form was sent to all students who participated in the session, and 110 (68.7%) students provided feedback. One forty-seven (91.9%) students were engaged during the online session, with 72% correct response during the first question, which gradually dropped to 56.2% as the session proceeded till the 12th (last) question at the end of one hour, with 53.3% responding correctly (Figure 1).

Eighty-seven percent of the students strongly agreed that the flipped classroom was interesting, the teaching material was good (84.2%), and the content was relevant (77.3%). Most students found the online session interactive and have acquired problem-solving abilities (Table 1). Most of them felt that the teaching method should be a part of the curriculum (Table 1).

**Table 1:** Student's perception of flipped classroom online module (n=110) to closed-ended questions [Likert 1: strongly disagree, 2: Disagree; 3: Neutral; 4: Agree; 5: Strongly agree].

Closed-ended responses	1	2	3	4	5	Mean (SD)	Satisfaction index
I found the flipped classroom interesting	0	0	3	11	96 (87.2%)	4.8 (0.4)	97.9
The quality of the teaching material was good	0	0	1	16	93 (84.5%)	4.8 (0.4)	97.1
The content of flipped module was relevant	0	0	2	23	85 (77.3%)	4.7 (0.5)	95.7
The faculty was well acquainted with the topic	0	0	0	3	107 (97.3%)	4.9 (0.2)	99.4
Session was interactive	0	0	1	14	95 (86.4%)	4.8 (0.4)	97.4
The time allotted for the flipped classroom was sufficient	1	3	14	29	63 (57.3%)	4.3 (0.8)	88.4
This module should be made part of the curriculum	1	0	7	16	86 (78.2%)	4.7 (0.7)	96.1
I feel I will be able to implement the knowledge I have learned	0	0	5	32	73 (66.4%)	4.6 (0.5)	93.9
Problem-solving ability has been inculcated	1	4	15	34	56 (50.9%)	4.1 (0.8)	89.5
I am now interested in the subject of Pediatrics	0	0	2	14	94 (85.5%)	4.8 (0.9)	97.4

The factors identified by the students that facilitated their learning include video-based questions and case-based teaching. The other factors included interactive teaching sessions, prior sharing of pre-recorded lectures, and a good teacher (Table 2). The barriers identified by students that hindered their learning during the session were categorized into technology-related (poor internet connectivity, app-related issues), teacher-related barriers (fast speed, language barrier), student/peer-related (frequent questions/chats), and few methodological barriers (inability to revise questions and difficulty in coordination) (Table 3). Students provided valuable suggestions to the teacher, including providing more resources, better time management, and better-quality video. A few technology-related suggestions included the choice of a better interactive app.

**Table 2:** Student's open-ended response to factors that facilitated their learning during flipped classroom session (n=110)

Student's illustrative quotes (few) on factors that facilitated learning	Emerged themes
Pre-recorded patient videos, along with questions based on videos, were beneficial, especially at this time when clinical postings are suspended	Use of video-based questions
The videos correlating to the conditions that we flip through the book	Use of interactive teaching
Videos give visual memory	Case-based teaching
Two-way interaction and little competition	Good teacher with effective time management
Interactive sessions along with cases to help understand better	Developed interest in the subject
Quick revision and implementation in various real-life case scenarios	Prior sharing of pre-recorded lecture
Question format rather than simply dictating as occurs in a regular class	
More time for cases	
Questions along with question bank	
Making the subject exciting and better retainment the subject	
The pre-recorded lecture helped us to have a Pre knowledge of the topic, so we had a better understanding of the topic	

**Table 3:** Student's open-ended response to factors that hindered their learning during flipped classroom session (n=110)

Student's illustrative quotes (few) on factors that hindered their learning	Emerged themes
Network issues: videos were stuttering	Technology (internet, app) barriers
Technical problems in the app hinder the participation of students	Teacher related barriers
In the Socrative app, even the correct answers were shown as incorrect	Student related barriers
the lecture was fast to grasp all	Methodological barriers
lack of deep knowledge in every topic	
teaching in the local language, content difficult to understand	
mentioning the multiple-choice answers in the app	
doubts of students in the chat box can hinder learning	
less student participation	
too many doubts distract the class	
students answering in the app	
use of two devices, one for class and the other for the app	
after the lecture, it wasn't easy to look back at the questions that I had missed	

Most faculty members agreed on the excellent quality of teaching (Satisfaction Index [SI]: 86.6), the relevance of content (SI: 86.6), and the appropriate TL methodology (SI: 93.3). All faculty agreed that time allotted for the session (SI: 80) was sufficient and that students were engaged during the session (SI: 80). All of them were neutral to students being less anxious (SI: 26.6) and less stressed (SI: 26.6) during the session. Most were motivated to use this TL method and found it satisfactory (SI: 93.3). There needed to be more open-ended faculty responses to draw meaningful conclusions.

## Discussion

The flipped classroom is a promising teaching approach that improves student engagement and increases learner motivation [7]. A systematic review of studies has shown that flipped classroom teaching is as effective as a traditional lecture regarding knowledge and skill gain [8]. In health professional education, meta-analysis has revealed that flipped classrooms were significantly more effective than traditional classroom sessions [6].

The three integral parts of flipped classroom sessions include pre-class preparation, in-class activity, and assessment of student learning [9]. The evaluation of student learning was incorporated in the in-class session, where the number of correct responses was recorded. The present study revealed that 92% of students were engaged in online classes. The engagement gradually dropped from the first question to the twelfth question. The drop in engagement could be attributed to the type of question, difficulty level of the question, loss of interest in the activity, or any technological difficulty like answering in the app and difficulty in coordination with the ongoing lecture as elicited from the students in open response. Engagement in an online class is a great challenge [10]. Students have always appreciated interactivity in large-group teaching as one of the factors which helped them learn [11].

The present study sensitized and motivated the faculty members on the flipped classroom teaching model, and the majority expressed adopting this teaching method. The study had limited faculty participation as the sessions were conducted during May-June 2020, when the COVID pandemic had just occurred. Various faculty were denoted with different COVID-related clinical and administrative responsibilities.

The students received utilization of classroom time on case scenarios/vignettes. Clinical videos, real-time patient scenarios, and problem-based teaching were additional factors that helped the students learn better during the session. The study results also need to be interpreted in the light of only 110 students responding to the feedback despite 160 students attending the online session. Students were reminded repeatedly and through personal contact with reminder emails and WhatsApp messages. Despite these efforts, during the COVID times, students could not be personally contacted for feedback; we acknowledge this as one of the study's limitations. The viewpoints of those 50 students who failed to respond to feedback could be precious as we presume they faced some issues or were probably not interested in the activity. It is well known that high self-motivation levels are required to implement the flipped classroom successfully [12].

Interestingly, despite the well-received flipped classroom session, many students acknowledged that it did not teach problem-solving skills. Some of the barriers identified by the students were technology-related, including poor internet connectivity, app-related issues, and difficulty in coordination between lecture and app, which has been reported by previous authors as well [13]. The students were encouraged to join the class using their laptop/desktop and answer the questions using the mobile app, but many had joined using mobile phones, which could have led to coordination issues. Some of the exciting barriers discovered in the study were frequent doubts from other peers, the teacher's language barrier, the teacher's fast pace, and less time devoted to the discussion of each case. Previous studies have demonstrated differences in faculty and students' perceptions of online teaching, with the former preferring synchronous education and the latter preferring pre-recorded lectures [14]. The current online flipped model serves as a blend of both. Students have expressed that flipped classroom neither improves nor worsens their sentiment toward online teaching [15]. Online flipped classroom models have been suggested as one way forward in online teaching and learning [16]. Limitations of the study include single-center and single-specialty experience. Many students should have responded to feedback despite attending the session, which may be considered while interpreting the results of the present study.

## Conclusions

The present study revealed that online flipped classroom teaching is feasible and acceptable to medical students and faculty, keeping the limitations of the present study in mind. Factors facilitated during the online flipped classroom model include interactive teaching sessions, prior sharing of pre-recorded lectures, and an excellent, enthusiastic teacher. Barriers to learning had poor internet connectivity, frequent interruptions from peers, and difficulty catching up with the teacher's pace. The introduction of flipped classroom strategy in an online model improved students' engagement, increasing their interest in the subject.

## Appendices

**Table 4:** Faculty Feedback Questionnaire

Closed-ended questions (Rate each answer on a scale of 1 to 5 where: 1: Strongly Disagree, 2: Disagree, 3: Neither agree nor disagree, 4: Agree, 5: Strongly Agree)	1	2	3	4	5
This module was need of the hour					
The quality of the teaching material was good					
The content of the flipped module was relevant					
The teaching-learning methodology was relevant to the topic					
The session was interactive					
The time allotted for the flipped classroom was sufficient					
Students were engaged during the session					
There was a change in the attitude of the students during the session					
Students were less anxious during the session					
Students were less stressed during the session					
Considering workload in the hospital, this module can be implemented					
Considering the time constraints, this module can be implemented					
This module can be used to cover some parts of the final year MBBS curriculum					
My overall experience with the module was satisfactory					
I am motivated to use this method of teaching in my future classes					
I feel I will be able to implement the module in my teaching					
Open ended questions					
What did you like the most in this module?					
What did you dislike in this module?					
What changes, if any, that will benefit the module?					
Any other comments and suggestions?					

**Table 5:** Student Feedback Questionnaire

Closed-ended questions (Rate each answer on a scale of 1 to 5 where: 1: Strongly Disagree, 2: Disagree, 3: Neither agree nor disagree, 4: Agree, 5: Strongly Agree)	1	2	3	4	5
I found flipped classroom method interesting and satisfactory					
The quality of the teaching material was good					
The content of the discussion was relevant					
The faculty was well acquainted with the topic					
Problem-solving approach has been taught to me after this session					
The session was interactive					
The time allotted was sufficient					
I feel I will be able to implement the knowledge I have learned					
Open-ended questions					
What were the factors which helped facilitate learning?					
What were the factors which helped facilitate learning?					
Any changes, if any, do you think would benefit the module?					
Any other comments and suggestions?					

## References

[1] Singh K, Mahajan R, Gupta P, Singh T (2018). Flipped classroom: a concept for engaging medical students in learning. Indian Pediatr.

[2] Gillispie V (2016). Using the flipped classroom to bridge the gap to generation Y. Ochsner J.

[3] Morgan H, McLean K, Chapman C, Fitzgerald J, Yousuf A, Hammoud M (2015). The flipped classroom for medical students. Clin Teach.

[4] Missildine K, Fountain R, Summers L, Gosselin K (2013). Flipping the classroom to improve student performance and satisfaction. J Nurs Educ.

[5] Tan E, Brainard A, Larkin GL (2015). Acceptability of the flipped classroom approach for in-house teaching in emergency medicine. Emerg Med Australas.

[6] Hew KF, Lo CK (2018). Flipped classroom improves student learning in health professions education: a meta-analysis. BMC Med Educ.

[7] Eaton M (2017). The flipped classroom. Clin Teach.

[8] Chen F, Lui AM, Martinelli SM (2017). A systematic review of the effectiveness of flipped classrooms in medical education. Med Educ.

[9] Persky AM, McLaughlin JE (2017). The flipped classroom - from theory to practice in health professional education. Am J Pharm Educ.

[10] Walsh LL, Arango-Caro S, Wester ER, Callis-Duehl K (2021). Training faculty as an institutional response to COVID-19 emergency remote teaching supported by data. CBE Life Sci Educ.

[11] Khan RA, Atta K, Sajjad M, Jawaid M (2022). Twelve tips to enhance student engagement in synchronous online teaching and learning. Med Teach.

[12] Lucardie AT, Berkenbosch L, van den Berg J, Busari JO (2017). Flipping the classroom to teach millennial residents medical leadership: a proof of concept. Adv Med Educ Pract.

[13] Li W, Gillies R, He M, Wu C, Liu S, Gong Z, Sun H (2021). Barriers and facilitators to online medical and nursing education during the COVID-19 pandemic: perspectives from international students from low- and middle-income countries and their teaching staff. Hum Resour Health.

[14] Watson C, Templet T, Leigh G, Broussard L, Gillis L (2023). Student and faculty perceptions of effectiveness of online teaching modalities. Nurse Educ Today.

[15] Lai VK (2021). Pandemic-driven online teaching-the natural setting for a flipped classroom?. J Biomech Eng.

[16] Babar S, Hidayat M (2022). Online flipped classroom (E-Fcr): way forward in Covid 19 era. J Ayub Med Coll Abbottabad.

